# Relationship between Energy Dosage and Apoptotic Cell Death by Modulated Electro-Hyperthermia

**DOI:** 10.1038/s41598-020-65823-2

**Published:** 2020-06-02

**Authors:** Patrick Hung-Ju Kao, Chia-Hung Chen, Yuk-Wah Tsang, Chen-Si Lin, Hsin-Chien Chiang, Cheng-Chung Huang, Mau-Shin Chi, Kai-Lin Yang, Wen-Tyng Li, Shang-Jyh Kao, Carrie Anne Minnaar, Kwan-Hwa Chi, Yu-Shan Wang

**Affiliations:** 1 Division of Cardiovascular Surgery, Department of Surgery, Shin Kong Wu Ho-Su Memorial Hospital, Taipei, Taiwan; 2 Institute of Molecular Medicine and Bioengineering, National Chiao Tung University, Hsinchu, Taiwan; 3 Department of Radiation Oncology, Chiayi Christian Hospital, Chiayi, Taiwan; 4 Department of Biomedical Engineering, Chung Yuan Christian University, Taoyuan, Taiwan; 5 Institute of Veterinary Science, School of Veterinary Medicine, National Taiwan University, Taipei, Taiwan; 6 Department of Radiation Therapy and Oncology, Shin Kong Wu Ho-Su Memorial Hospital, Taipei, Taiwan; 7 Department of Biochemistry and Molecular Biology, College of Medicine, National Taiwan University, Taipei, Taiwan; 8 Institute of Radiation Science and School of Medicine, National Yang-Ming University, Taipei, Taiwan; 9 Division of Chest Medicine, Shin Kong Wu Ho-Su Memorial Hospital, Taipei, Taiwan; 10 Department of Radiation Sciences, University of the Witwatersrand, Johannesburg, South Africa

**Keywords:** Cancer, Cancer therapy

## Abstract

Modulated electro-hyperthermia (mEHT) is a form of mild hyperthermia (HT) used for cancer treatment. The principle utility of HT is the ability not only to increase cell temperature, but also to increase blood flow and associated pO_2_ to the microenvironment. While investigational evidence has shown the unique ability of mEHT to elicit apoptosis in cancer cells, *in vivo* and *in vitro*, the same trait has not been observed with conventional HT. There is dissension as to what allows mEHT to elicit apoptosis despite heating to only mild temperatures, with the predominant opinion in favor of increased temperature at a cellular level as the driving force. For this study, we hypothesized that in addition to temperature, the amount of electrical energy delivered is a major factor in induction of apoptosis by mEHT. To evaluate the impact of electrical energy on apoptosis, we divided generally practiced mEHT treatment into 3 phases: Phase I (treatment start to 10 min. mark): escalation from 25 °C to 37 °C Phase II (10 min. mark to 15 min. mark): escalation from 37 °C to 42 °C Phase III (15 min. mark to 45 min. mark): maintenance at 42 °C Combinations of mEHT at 18 W power, mEHT at 7.5 W power, water bath, and incubator were applied to each of the three phases. Power output was recorded per second and calculated as average power per second. Total number of corresponding Joules emitted per each experiment was also recorded. The biological effect of apoptotic cell death was assayed by annexin-V assay. In group where mEHT was applied for all three phases, apoptosis rate was measured at 31.18 ± 1.47%. In group where mEHT was only applied in Phases II and III, apoptosis rate dropped to 20.2 ± 2.1%. Where mEHT was only applied in Phase III, apoptosis was 6.4 ± 1.7%. Interestingly, when mEHT was applied in Phases I and II, whether Phase III was conducted in either water bath at 42 °C or incubator at 37 °C, resulted in nearly identical apoptosis rates, 26 ± 4.4% and 25.9 ± 3.1%, respectively. These results showed that accumulation of mEHT at high-powered setting (18 W/sec) during temperature escalation (Phase I and Phase II), significantly increased apoptosis of tested cancer cells. The data also showed that whereas apoptosis rate was significantly increased during temperature escalation by higher power (18 W/sec), apoptosis was limited during temperature maintenance with lower power (7.5 W/sec). This presents that neither maintenance of 42 °C nor accumulation of Joules by mEHT has immediate correlating effect on apoptosis rate. These findings may offer a basis for direction of clinical application of mEHT treatment.

## Introduction

Hyperthermia (HT) is a method of cancer treatment in which patients are subjected to supra-normal body temperatures, and is often used in conjunction with radiotherapy or chemotherapy^1,2^. Modulated electro-hyperthermia (mEHT) is a loco-regional hyperthermia method utilizing electromagnetic current at 13.56 MHz radiofrequency (RF) to treat tumours^3–5^. Modulated electro-hyperthermia is applied for cancer treatment in numerous countries, including those of the European Union, Australia, Canada, Israel, Russia, South Africa, South Korea, Taiwan, and Thailand, with a history of clinical practice spanning two decades^6–8^. The tradename of mEHT is ‘Oncothermia.’

Heating techniques utilize either a capacitive or phased array heating system. Capacitive heating technologies rely on the difference in the impedance and conductivity of tumours, resulting from the increased metabolic activity of the tumour, the subsequently higher ionic concentration within the extracellular tumour environment, and the cellular disorganization^4,5,9,10^. Modulated electro-hyperthermia utilizes a capacitive coupled set-up with additional safety parameters and impedance matching to further improve the selection of the tumours over the healthy tissue and to reduce the risks of hot spots and superficial burns^9–11^. Cell membranes contain sterol- and sphingolipid-enriched domains which have different biophysical properties, such as conductivity, to the rest of the cell membrane. These domains are subsequently more sensitive to the absorption of the applied energy and simulations indicate that this induces increased temperatures on a nanoscopic level^12–14^. This nanoscale heating stimulates signal transduction pathways and involved in the programmed cell death in cancer cell^4,15^. The ability of mEHT to induce cancer cell apoptosis was reported in several studies^14,16,17^. A direct comparison between water bath, conventional hyperthermia (Thermotron RF-8, 8 MHz), and mEHT for their biological effects has been previously published^18^. Under the same temperature conditions (42 °C for 30 min), mEHT could induce significantly higher apoptosis rates than water bath and conventional hyperthermia. The apoptotic rate of mEHT at 42 °C was similar to water bath at temperatures between 45 °C and 48 °C (approximately 46 °C)^18^. The hypothesis on the mechanism of mEHT for cancer cell apoptosis is stimulation of the cell membrane and activation of death receptor on cell membranes to induce apoptosis. Andocs *et al*. have previously investigated the apoptotic mechanism of mEHT treatment. The protein expression of cell death receptor, Fas, markedly increased 3 h after mEHT treatment, compared to treatment using alternate hyperthermia methods. Increased activation of the enzyme Caspase-8 was observed 1 h and 3 h following mEHT treatment, compared to alternate hyperthermia methods^14^. This suggests that mEHT can induce more apoptotic cell death than other hyperthermia techniques, possibly due to the additional effect of energy disposition.

Several studies have explored the mechanisms of mEHT^3–5,9,10,15,19,20^. Clinical reports for mEHT treatment indicate feasibility and benefit for cancer patients with relapsed malignant glioma^7,21^, advanced hepatocellular carcinoma^22,23^, and advanced colorectal cancer with liver metastasis^24^, amongst others. In clinical practice, mEHT treatment is performed on patients from one to three sessions per week. Each treatment session is upwards of 60 minutes, with maximum power being 150 W. Power is regularly and linearly elevated depending on individual patient tolerance. The applicators used are either 20 cm or 30 cm in diameter, depending on the tumour volume and treatment area. Typically, hyperthermia techniques attempt to measure temperatures inside the tumour, using the temperature as a measurement of treatment efficiency. Challenges associated with accurate intra-tumoural temperature measurements led to the proposal of a different dosing method utilized during mEHT. During mEHT, the energy deposition is measured as a dose of the treatment, and the temperature is only an outcome of the effects of the treatment, but not the driving force of the treatment. The lower temperatures achieved during mEHT are associated with improved safety^25^. Temperature is therefore not measured during mEHT treatments in clinical practice^12^. New studies in mEHT have centered around the theory that the total energy absorbed by the subject (measured in Joules) is a principal factor in cancer treatment, rather than the transfer of thermal energy^26,27^. The average applied energy is around 300 kilojoule per treatment (range: 250 to 450). The clinical application does not increase the energy power more than what the patient can tolerate. In some case, the treatment parameters do not even achieve the defined values. This may be the reason for the varied clinical results compared to laboratory results. In this study, we hypothesized that if the patient can tolerate a higher power, or more total energy, an increased therapeutic outcome may be achieved.

Since mEHT treatment induces a significant increase in cell apoptosis at the same temperature as other heating techniques^15^, we hypothesized that the positive results of mEHT treatments are not in fact due to the direct effect of hyperthermia on the cell signaling processes, but rather due to the effects mediated by the electric fields delivered to tumour tissues. To test this hypothesis, we investigated which parameters are the most important factors affecting the mEHT-induced apoptotic cell death effect on cancer cells. This may assist in the optimization and improvement of clinical results to match prior laboratory observations. We designed several experimental set-ups to investigate whether differences in the accumulation of total Joules and power wattage per second with modulation would induce different levels of apoptosis on the human lung cancer cell line, A549. The maintenance of 42 °C, accumulation of total Joules, and accumulation of high or low power wattage per second with modulation, were analyzed.

## Materials and Methods

### Cell culture

The adenocarcinomic human alveolar basal epithelial cell line, A549, was maintained in Dulbecco’s Modified Eagle Medium (DMEM; Invitrogen, Verviers, Belgium) containing 10% heat-inactivated fetal bovine serum (FBS), 2 mM L-glutamine, 100 units/mL penicillin, and 100 µg/mL streptomycin (SIGMA, St. Louis, MO). A549 was purchased from BCRC (Bioresource Collection and Research Center, Hsinchu, Taiwan, catalog number BCRC 60074).

### mEHT treatment

Electromagnetic heating was generated by capacitively-coupled, amplitude-modulated, 13.56-MHz radiofrequency (LabEHY, Oncotherm GmbH, Troisdorf, Germany). An *in vitro* heating model was set up in an electrode chamber (LabEHY *in vitro* applicator) as previously described^18^. The chamber contained a cell bag (1 × 10^6^ cells) heated from room temperature (25 °C) to 42 °C with average power of 18 W, and then maintained at 42 °C for 30 minutes, with average power of 7.5 W. For the groups that required mEHT at any experimental phase, the cells were transferred at start of experiment to a cell bag, and remained in the same bag for the subsequent phases. The temperature was measured using optical sensors (Luxtron FOT Lab Kit, LumaSense Technologies, Inc., California, USA). The power pattern was checked each time to verify the accuracy and similarity of the experiments. The mEHT treatment was divided into three phases to evaluate the impact of electrical energy on apoptosis:

Phase I (treatment start to 10 min. mark): escalation from 25 °C to 37 °C

Phase II (10 min. mark to 15 min. mark): escalation from 37 °C to 42 °C

Phase III (15 min. mark to 45 min. mark): maintenance at 42 °C

Experiments were conducted with combinations of mEHT at 18 W power (to increase temperature) and mEHT at 7.5 W power (to maintain temperature). For mEHT, power watt output was recorded per second, and calculated as average power per second and total Joule (watt x second).

### Water bath and incubator heating

Two control experiments were set up, with both water bath heating and incubator heating applied to each of the three phases as control comparisons. For negative control, cells were transferred to a tube, and subsequently incubated at 37 °C for a period equal to the combined time of Phases I, II, and III (45 mins.)

A second group was set up as ‘temperature control,’ with the purpose to mimic temperature-reached over time of the mEHT experimental groups. At start, cells were transferred to tube, and placed in water bath for Phase I heating, from 25 °C to 37 °C over a period of 10 minutes. Phase II immediately followed, with cells heated from 37 °C to 42 °C over a period of 5 minutes. This was then immediately followed by Phase III, with temperature of water bath maintained at 42 °C for 30 minutes.

### Apoptosis assay

A549 cells (1 × 10^6^ cells) were treated by combinations of mEHT and/or water bath, seeded on 6-well plates and cultured for 24 hours, trypsinized, and washed twice with phosphate-buffered saline (PBS). Apoptosis was confirmed using an Annexin-V Apoptosis Kit (BD Pharmingen) according to manufacturer instructions. Briefly, tumour cells were washed three times with PBS, stained with Annexin V and propidium iodide, incubated in the dark and on ice for 10 minutes, and then subjected to flow cytometry analysis. The percentage of positive cells was measured using a FACSCalibur cytometer and Cell Quest Pro software (Becton Dickinson, Mountain View, CA). The apoptosis rates induced by mEHT of each experiment were evaluated individually via Annexin-V assay to confirm the energy of mEHT was delivered to cells and induced proper cell death effect successfully.

### Statistical analysis

All results were compared using an unpaired *t*-test (2-tailed). Differences were considered statistically significant at a P-value <0.05. Data were represented as the mean ± SD (standard deviation). Pearson’s correlation coefficient was determined to assess linear relationship between power wattage output and apoptotic cell death in Graphpad prism 6.

## Results

### Round 1: different total joules induced different levels of apoptotic cell death

**Figure 1:**
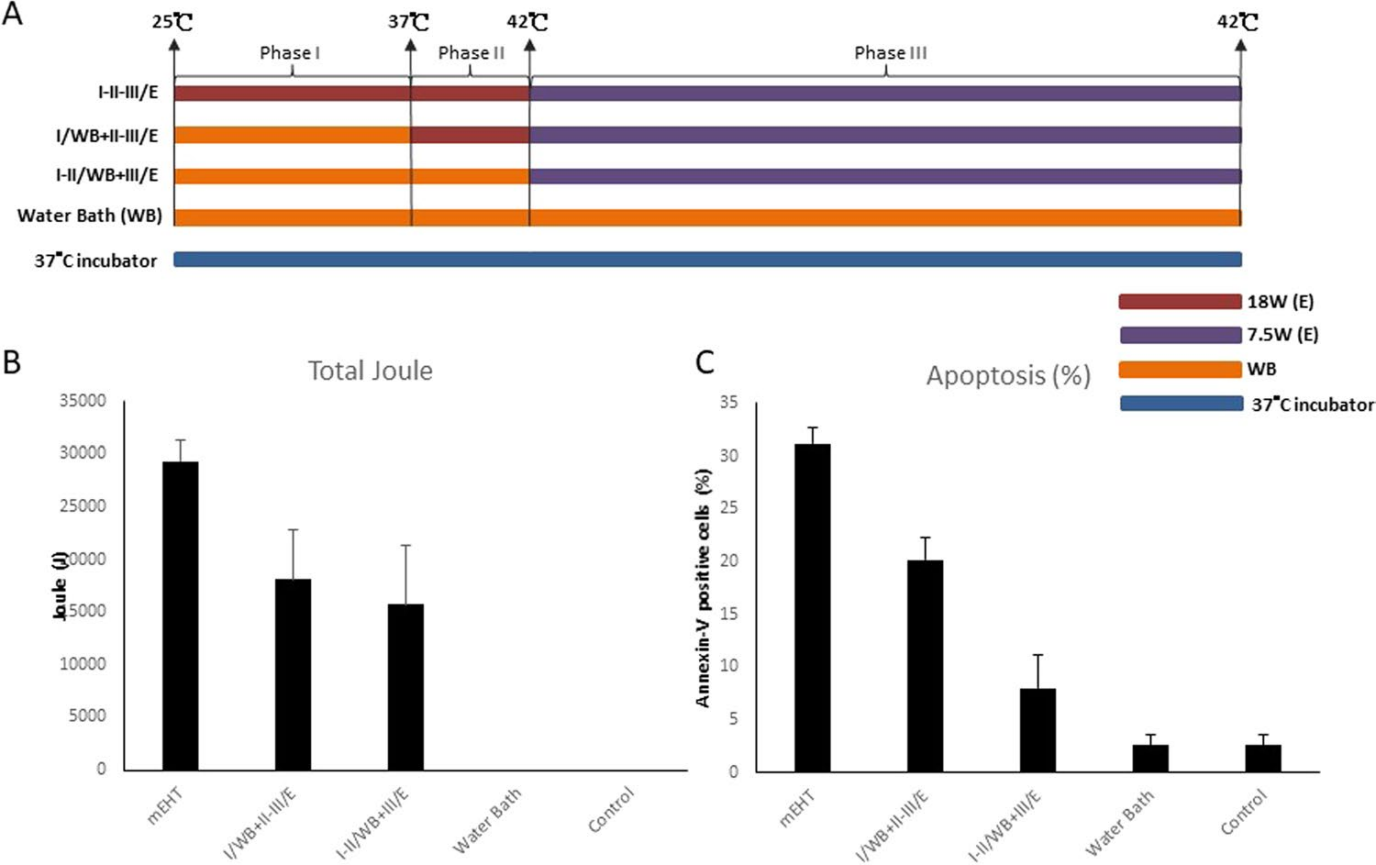
Evaluation of Temperature Increase Phase. (**A**) The heating protocol has three phases and a set of variation, fulfilled with three variant of energy-absorption (1) electric (mEHT), water-bath (WB, max.42 °C), (3) incubator (constant 37 °C). The incubator had fixed temperature the WB was controlled. Hyperthermia treatment was divided into three phases to evaluate the impact of electrical energy on apoptosis: Phase I) Escalation from room temperature to 37 °C in span of 10 minutes. Phase II) Escalation from 37 °C to 42 °C in span of 5 minutes. Phase III) Maintenance of 42 °C for 30 minutes. I/WB + II-III/E: Phase I was heated by WB and Phase II and III were heated by mEHT. I-II/WB + III/E: Phase I and II were heated by WB and Phase III was heated by mEHT. (**B**) The absorbed energy was measured only in case of electric energy transfer. Power of mEHT was setting as Phase I, II-18W and Phase III-7.5 W. Power output was recorded per second and calculated as average power per second. Total number of corresponding Joules emitted per each experiment was recorded and showed as bar chart. (**C**) Apoptosis was measured using flow cytometry after staining with FITC-conjugated Annexin V and propidium iodide. Positively stained cells were counted using FACSCalibur. Histograms for the percentage of Annexin-V-positive cells are shown. Data represent results from 3 independent experiments; bars indicate mean ± standard deviation (SD). (*P < 0.05).

To evaluate the impact of electrical energy on apoptosis, we wanted to test heating by mEHT 18 W versus heating by water bath. Treatment time of 45 minutes was divided into three phases (Fig. 1A):

Phase I (treatment start to 10 min. mark): escalation from 25 °C to 37 °C

Phase II (10 min. mark to 15 min. mark): escalation from 37 °C to 42 °C

Phase III (15 min. mark to 45 min. mark): maintenance at 42 °C

Apoptotic cell death was evaluated using FITC-conjugated Annexin V and propidium iodide 24 h after treatment.

In the first group (I-II-III/E), Phase I utilized 18 W power of mEHT to increase temperature from 25 °C to 37 °C over span of 10 minutes. In Phase II, settings remained unchanged to increase temperature from 37  °C to 42 °C over span of 5 minutes. In Phase III, 7.5 W power of mEHT was then used to maintain temperature at 42 °C for 30 minutes. The total power of mEHT absorbed was 29317.7 ± 1984.7 Joules (Fig. 1B).

In the second group (I/WB + II-III/E), Phase I heating was substituted by water bath heating, resulting in total Joules absorbed to be 18125.7 ± 4717.1 deposited by mEHT during Phases II and III (Fig. 1B).

In the third group (I-II/WB + III/E), both Phase I and II utilized water bath heating, while Phase III remained mEHT 7.5 W, and total Joules absorbed was recorded as 15724.8 ± 5581.4 deposited by mEHT during Phase III (Fig. 1B).

In the fourth group (WB), Phases I, II, and III were conducted on cells using water bath. In Phase I, water bath was used to increase temperature from 25 °C to 37 °C over span of 10 minutes. In Phase II, water bath was used to increase temperature from 37 °C to 42 °C over span of 5 minutes. In Phase III, water bath of 42 °C was used to maintain temperature for 30 minutes. As mEHT was not utilized, 0 Joules were absorbed (Fig. 1B).

A fifth group (37 °C incubator) was set as control, replacing Phases I, II, and III, with incubation at 37 °C for a total of 45 minutes.

Results from these experiments showed a significantly higher proportion of apoptotic cells in the first group (I-II-III/E) (31.18 ± 1.47%) compared with the group (WB) where only water bath was used for heating. Interestingly, the control group (37 °C incubator) had apoptosis rates comparable to water bath group (WB).

The (I/WB + II-III/E) group, where Phase I used water bath heating, apoptosis was significantly less than (I-II-III/E), 22.6 ± 4.42% versus 31.18 ± 1.47%, respectively (Fig. 1C). Although tumour cells were subjected to same temperatures and same durations, cell death by apoptosis showed positive correlation to accumulation of power wattage. These results indicate that mEHT treatment conducted at various total Joule-count would result in differing levels of apoptotic cell death.

### Round 2: limited role of temperature maintenance at 42° in apoptosis

We wanted to investigate the role of 42 °C maintenance phase (Phase III) in cell death by apoptosis. Using the first group (I-II-III/E) from Round 1 as the baseline, where Phase I utilized 18 W power of mEHT to increase temperature from 25 °C to 37 °C over span of 10 minutes, Phase II utilized 18 W power of mEHT to increase temperature from 37 °C to 42 °C over span of 5 minutes, and then Phase III utilizes 7.5 W power of mEHT to maintain temperature at 42 °C for 30 minutes. The total power of mEHT absorbed was 29317.7 ± 1984.7 Joules and apoptosis was 31.18 ± 1.47%).

**Figure 2:**
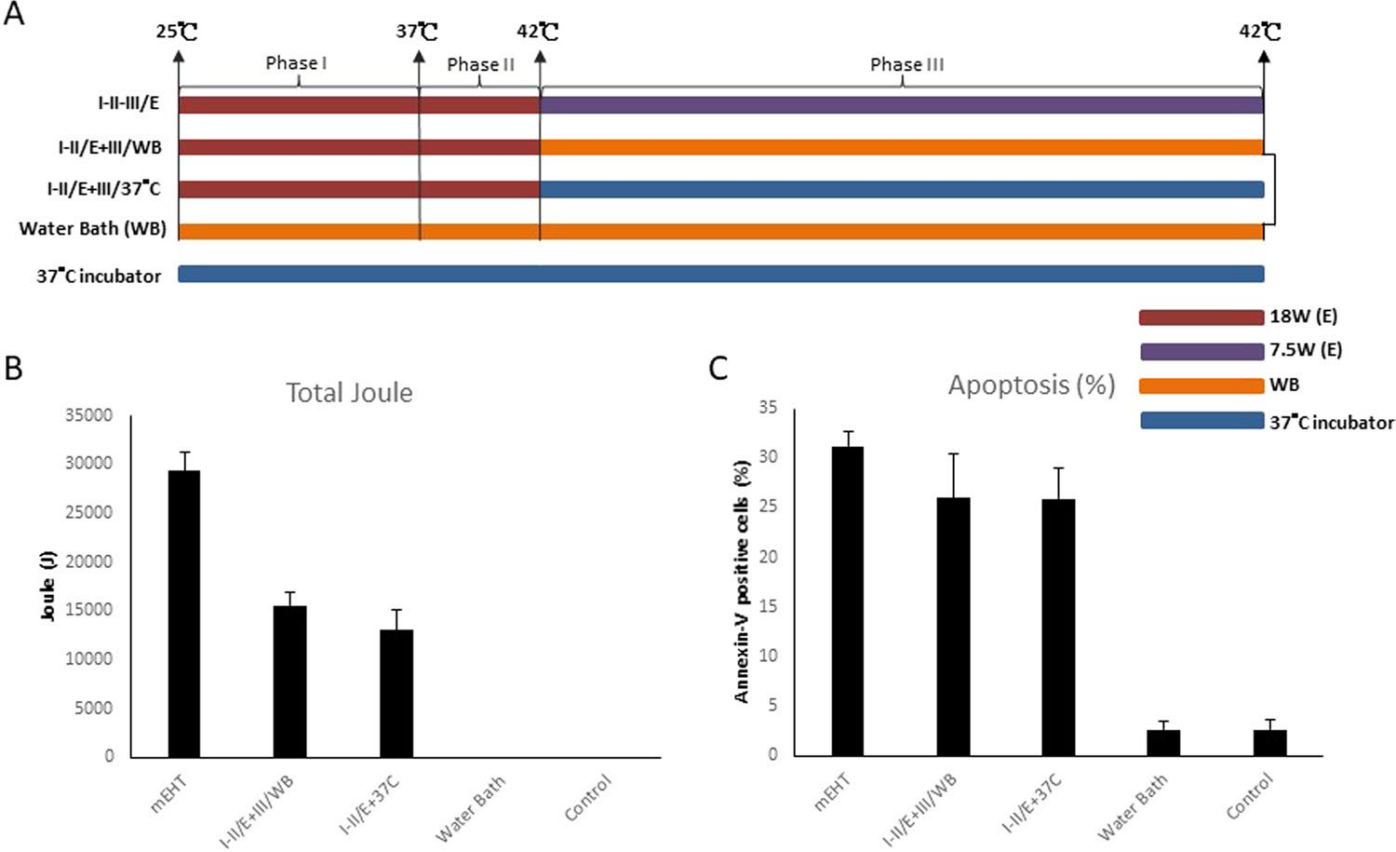
Evaluation of Phase III Temperature. (**A**) The heating protocols. I-II/E + III/WB: Phase I and II were heated by mEHT and Phase III was heated by WB. I-II/E + III/37 °C: Phase I and II were heated by mEHT and Phase III was shifted to 37 °C incubator. (**B**) Total number of corresponding Joules emitted per each experiment was recorded and showed as bar chart as previous figure described. (**C**) Apoptosis was measured as previous figure described. Histograms for the percentage of Annexin-V-positive cells are shown. Data represent results from 3 independent experiments; bars indicate mean ± standard deviation (SD). (*P < 0.05).

Experiments followed the same segmentation as those from Round 1 (Fig. 2A):

Phase I (treatment start to 10 min. mark): escalation from 25 °C to 37 °C

Phase II (10 min. mark to 15 min. mark): escalation from 37 °C to 42 °C

Phase III (15 min. mark to 45 min. mark): maintenance at 42 °C

The second group (I-II/E + III/WB) replaced maintenance of 42 °C (Phase III) of 7.5 W power of mEHT with maintenance in 42 °C water bath. The total Joules absorbed decreased to 16203.2 ± 605.6 (Fig. 2B).

The third group (I-II/E + III/ 37 °C) replaced maintenance of 42 °C (Phase III) of 7.5 W power of mEHT with maintenance in 37 °C incubator. The total Joules absorbed decreased to 14924.5 ± 2431.8 (Fig. 2B). The total Joules were accounted only from mEHT treatment which accumulated power per second for total treatment time.

Interestingly, although the total Joules absorbed were significantly different amongst the three experiment groups, the apoptosis rates were not significantly different. Whereas the baseline group (I-II-III/E) had apoptosis at 31.18 ± 1.47%, the second group (I-II/E + III/WB) had apoptosis of 26 ± 4.4%, and the third group (I-II/E + III/ 37 °C) had apoptosis of 25.9 ± 3.1% (Fig. 2C). These results indicate limited apoptosis induction ability of temperature maintenance (Phase III), once temperatures reach 42 °C.

### Round 3: pivotal role of high-power mEHT in the induction of apoptotic cell death

We further evaluated the effects of 18 W power on apoptotic cell death. Once again, we kept the first group (I-II-III/E) from Round 1 as the baseline, where Phase I utilized 18 W power of mEHT to increase temperature from 25 °C to 37 °C over span of 10 minutes, Phase II utilized 18 W power of mEHT to increase temperature from 37 °C to 42 °C over span of 5 minutes, and then Phase III utilizes 7.5W power of mEHT to maintain temperature at 42 °C for 30 minutes. The total power of mEHT absorbed was 29317.7 ± 1984.7 Joules and apoptosis was 31.18 ± 1.47%).

**Figure 3:**
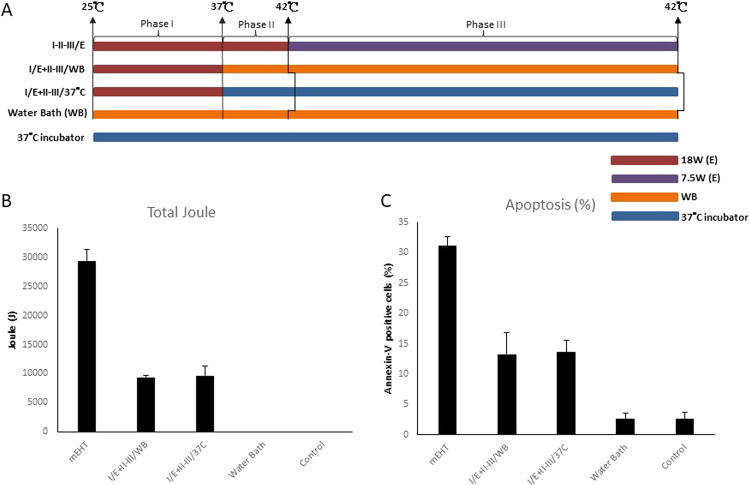
Evaluation of Phase II Temperature Increase: The first temperature increasing phase (Phase I, 25 °C → 37 °C) could cause significant elevation of apoptotic rate. (**A**) The heating protocols. I/E + II-III/WB: Phase I was heated by mEHT and Phase II and III were heated by WB. I/E + II-III/37 °C: Phase I was heated by mEHT and Phase II and III were shifted to 37 °C incubator. (**B**) Total number of corresponding Joules emitted per each experiment was recorded and shown as bar chart as previous figure described. (**C**) Apoptosis was measured as previous figure described. Histograms for the percentage of Annexin-V-positive cells are shown. Data represent results from 3 independent experiments; bars indicate mean ± standard deviation (SD). (*P < 0.05).

Experiments followed the same segmentation as previous rounds (Fig. 3A):

Phase I (treatment start to 10 min. mark): escalation from 25 °C to 37 °C

Phase II (10 min. mark to 15 min. mark): escalation from 37 °C to 42 °C

Phase III (15 min. mark to 45 min. mark): maintenance at 42 °C

Experiments on the second group (I/E + II-III/WB) utilized water bath heating in Phase II to increase temperature from 37 °C to 42 °C over span of 5 minutes, and water bath maintenance in Phase III to maintain temperature at 42 °C for 30 minutes. Total Joules absorbed were to 9318.6 ± 371 (Fig. 3B).

The third group (I/E + II-III/37 °C) utilized maintenance in 37 °C incubator for Phases II and III. The total Joules absorbed decreased from the first group to a similar total as the second group to 9637.1 ± 1685.3 (Fig. 3B).

Minute differences in apoptotic cell death were observed between the water bath heating group (I/E + II-III/WB) and the 37 °C incubator group, 13.52 ± 2.6% versus 13.6 ± 2%, respectively (Fig. 3C). However, these rates were significantly lower than the baseline group (I-II-III/E) of 31.18 ± 1.47%. In contrast, the (I/WB + II-III/E) group from Round 1, where Phase I utilized water bath heating from 25 °C to 37 °C, followed by Phase II with 18 W mEHT, had apoptosis of 22.6 ± 4.42% (Fig. 1C). Thereby, apoptosis effect by mEHT heating from 37 °C to 42 °C during short span of 5 minutes, shows significant value and efficiency, and warrants further exploration.

### Round 4: comparison of low-power and high-power mEHT

To follow up on our findings from Round 3, we wished to further investigate whether high-powered heating-induced apoptosis was caused by the accumulation of total Joules or by temperature increase over time.

**Figure 4:**
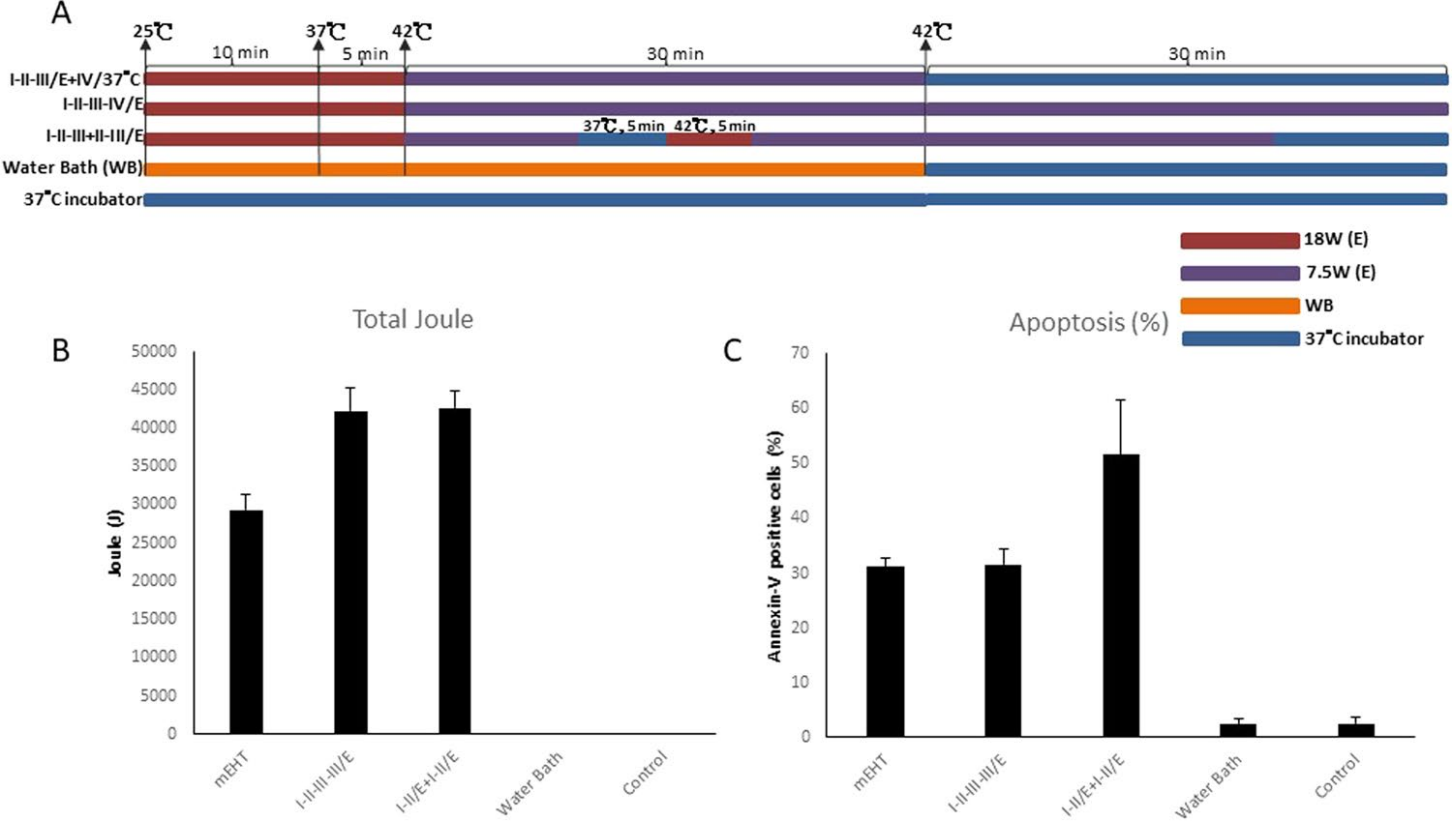
Repeating Phase II vs. Lengthening Phase III: Two rounds of temperature increasing phase and one round or elongation maintenance phase. (**A**) The heating protocols. I/E + II-III/WB: Phase I was heated by mEHT and Phase II and III were heated by WB. I/E + II-III/37 °C: Phase I was heated by mEHT and Phase II and III were shifted to 37 °C incubator. (**B**) Total number of corresponding Joules emitted per each experiment was recorded and shown as bar chart as previous figure described. (**C**) Apoptosis was measured as previous figure described. Histograms for the percentage of Annexin-V-positive cells are shown. Data represent results from 3 independent experiments; bars indicate mean ± standard deviation (SD). (*P < 0.05).

For this experiment, treatment time of 75 minutes was divided into four phases using various settings of heating (Fig. 4A):

Phase I (treatment start to 10 min. mark): escalation from 25 °C to 37 °C

Phase II (10 min. mark to 15 min. mark): escalation from 37 °C to 42 °C

Phase III (15 min. mark to 45 min. mark): variable

Another round of Phase III (45 min. mark to 75 min. mark): maintenance at 42 °C

As with previous rounds, we kept the first group (I-II-III/E) from Round 1 as the baseline, where Phase I utilized 18 W power of mEHT to increase temperature from 25 °C to 37 °C over span of 10 minutes, Phase II utilized 18 W power of mEHT to increase temperature from 37°C to 42°C over span of 5 minutes, and then Phase III utilizes 7.5 W power of mEHT to maintain temperature at 42 °C for 30 minutes.

In the second group (I-II-III-III/E), 18 W power of mEHT was used to increase temperature from 25 °C to 42 °C, then 7.5 W power of mEHT was used to maintain temperature at 42 °C for 60 minutes. The results of the second group (I-II-III-III/E) did not show increased apoptotic cell death (31.63 ± 2.75%) when compared with baseline group from Round 1 (I-II-III/E) (31.18 ± 1.47%), even though the total Joules accumulated were significantly greater (42262.6 ± 2932.5 versus 29317.7 ± 1984.7, respectively) (Fig. 4C).

In the third group (I-II +I- II-III/E), 18 W power of mEHT was used to increase temperature from 25 °C to 42 °C, then 7.5 W power of mEHT was used to maintain temperature at 42 °C for 10 minutes, then cooled to 25 °C by water bath, then again treated with 18 W power of mEHT to increase temperature to 42 °C, then 7.5 W power of mEHT was used to maintain temperature at 42 °C by 7.5 W for 30 minutes.

Although the total Joules accumulated by the second group (I-II-III-III/E) and third group (I-II-III + II-III/E) were similar, 42262.6 ± 2932.5 and 43264.2 ± 2862.7, respectively (Fig. 4B), the third group had significantly higher apoptosis (51.6 ± 9.85%). Whereas (I-II-III-III/E) had maintenance of 42 °C with 7.5 W power of mEHT for Phase III and Phase IV, we experimented by adding an additional cooling and heating segment to the (I-II-III + II-III/E) group; Phase III started with 10 minutes of 7.5 W mEHT, followed by 5 minutes of 37 °C incubator, followed by another 5 minutes of 18 W mEHT to reach 42 °C a second time, followed by 30 minutes of 7.5 W mEHT to maintain 42 °C, followed with 10 minutes of 37 °C incubator.

These results show that low-power heating by mEHT did not induce apoptosis as effectively as of high-power heating, with time and accumulated Joules being equal. The results also corroborated our findings from Round 3, where 18 W power of mEHT for 5 minutes was found to be most significant setting for apoptosis. By doubling this setting, apoptosis rates drastically increased to 51.6 ± 9.85%, dwarfing the apoptosis rates from the previous settings.

### Correlation of power levels and apoptotic cell death

**Figure 5:**
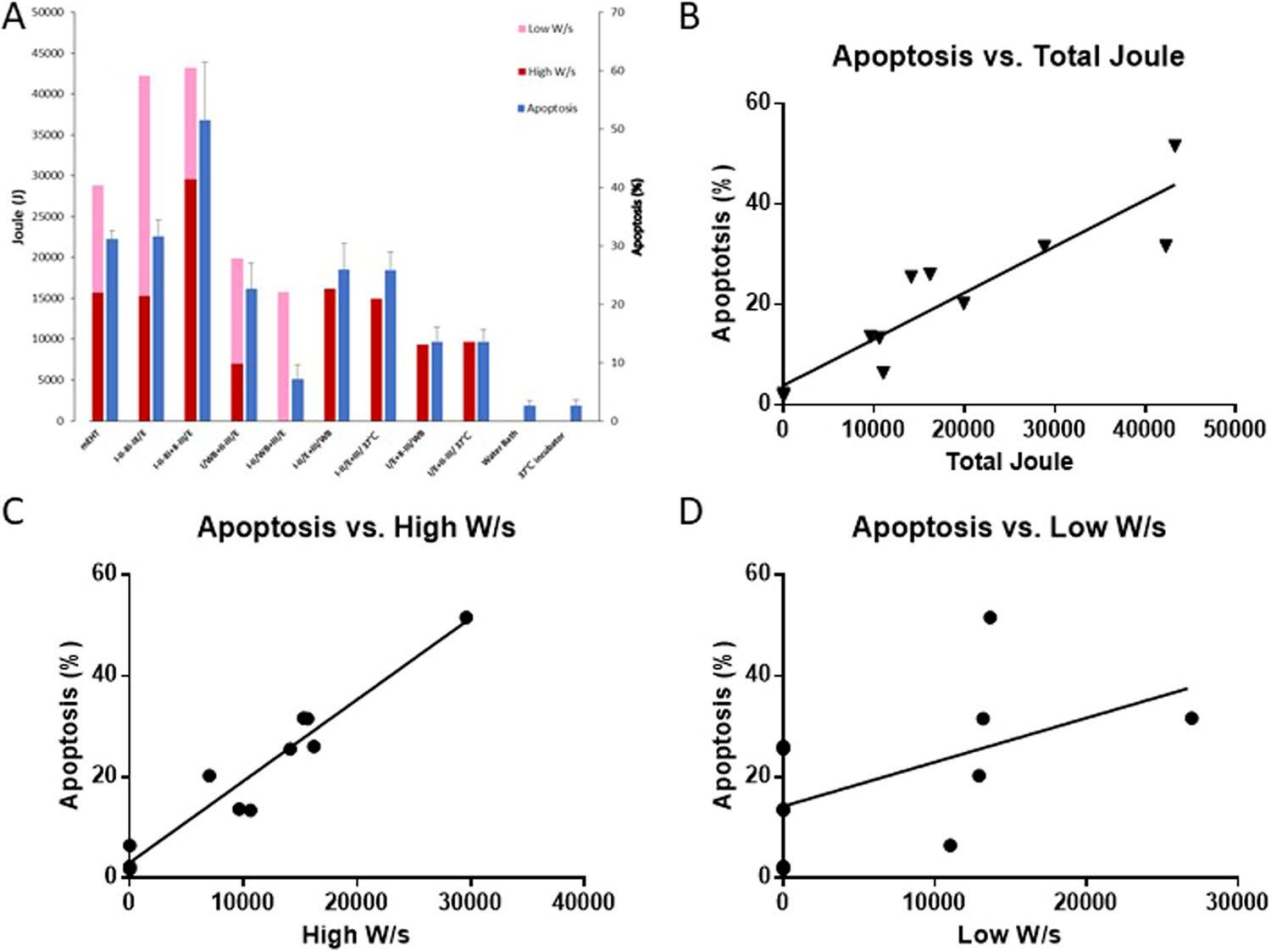
Correlation of Watt/sec and Apoptosis Rate: The SAR/c values well characterized the dose of the treatment. (**A**) The comparison of total number of corresponding Joules emitted per each experiment and apoptotic rate was showed as bar chart. (**B**) Relation between the total Joules (x-axis) and apoptotic rate (y-axis). Representative of three experiments. (**C**) Relation between the high watt (x-axis) and apoptotic rate (y-axis). Representative of three experiments. P-values in F represent the probability that there is not a negative correlation between high watt and apoptotic rate. (**D**) Relation between the low watt (x-axis) and apoptotic rate (y-axis). Representative of three experiments.

The direct comparison of total accumulated Joules at high-power and low-power levels to apoptotic cell death percentage is shown in Fig. 5A. Correlation analysis was performed to determine power levels to apoptotic cell percentage. The correlation between total accumulated Joules and induction of apoptotic cell death was shown to have significant positive correlation (r = 0.9106, p < 0.0001, Fig. 5B). There was also a high degree of significant positive correlation (r = 0.9649) between high-power heating (18 W) and induction of apoptotic cell death (p < 0.0001, Fig. 5C). Conversely, a low degree of positive correlation was found between low-power heating (7.5 W) and induction of apoptotic cell death, which did not reach statistical significance (r = 0.5300, p = 0.0935, Fig. 5D). Low-power heating (7.5 W) and induction of apoptotic cell death did not show positive correlation.

## Discussion

For many years, the study of hyperthermia was focused mainly on the effect of increased temperatures. However, the hyperthermia technology for deep site heating has improved over time. The additional effect of electronic or magnetic-based devices used to conduct deep site heating was ignored. In this study, we found that the accumulation of relatively high-power wattage per second (18 W/sec) during temperature escalation (Phase I and Phase II), significantly induced apoptotic death in cancer cells. The data also showed that whereas apoptosis rate was significantly increased during temperature escalation (18 W/sec), apoptosis was limited during temperature maintenance (7.5 W/sec). This presents that neither the maintenance of 42 °C nor the accumulation of Joules by mEHT may have mediated correlating effect on apoptosis. Water bath heating at 42 °C did not show an ability to significantly induce apoptosis, with time being constant, which matches our previously observations^18^. In that previous study, we had found other commercially available hyperthermia devices to have insignificant effects on apoptosis^18^.

The reducing the temperature of cell cultures to the relative harsh environment of 25 °C may cause lower proliferation, modified differentiation, and protein production. In order to prevent this misreading, we used a dual control setup. In the first control group, we heated cells in water bath starting at 25 °C, mimicking the temperature increase of the mEHT treatment groups, to isolate temperature-dependent results. To guard against the chance that lowering of temperatures to 25 °C affected outcomes, the second control group was setup, whereby all cells were placed in test tube and maintained at 37 °C throughout all phases of testing. While this experiment design may be artificial, the observations on cellular apoptosis showed stark contrast in the five groups. The results point toward apoptosis causing effects of mEHT to be temperature-independent. Our previous studies have shown thermal effects of mEHT, particularly in *in vivo* models, where the elevation of temperature during mEHT treatment induces the up-regulation of heat shock protein, stimulating intra-tumoural inflammatory cytokine-expression and synergistic effects with dendritic cell therapy^16^. We believe these effects to be caused by the elevation of temperature, and the incorporation of both non-thermal and thermal effects by mEHT in cancer treatment.

There are numerous heating methods in clinical use, but not all methods have shown beneficial results. Hyperthermia by phased array systems showed benefit combined with radiotherapy for cervical cancer as investigated by van der Zee^28^, and capacitive heating failed to show a benefit in a phase three trial combined with CCRT for cervical cancer published by Harima^29^. In the results of our present study, round 1 shows a higher importance of the energy deposition than of temperature, on the rates of apoptosis. It is important to note that this does not negate the effects of temperature on the sensitization of tumours to treatments such as radiotherapy and chemotherapy, as applied in conventional hyperthermia. These effects of hyperthermia are well known. However, the effects of mEHT, which has a milder heating effect, as a sensitizer are being demonstrated in studies such as that by Minnaar *et al*.^30^. Modulated electro-hyperthermia has also shown to improve disease management in palliative treatment for brain and pancreatic tumours as a standalone treatment^31,32^. The clinical results showing mEHT has an effect in the absence of higher temperatures prompted the research group to investigate other effects of the technique.

The beneficial impact of this thermal effect is both a fever-range temperature-mediated local cellular response and a systemic physiological response transferred via the blood to the rest of the body. Additionally, mEHT induces greater biological effects than conventional forms of hyperthermia. In mEHT treatment, the major role of temperature surveillance was to avoid any sort of damage during therapy. This study showed that the energy disposition was associated with the highest rates of apoptosis. This study is investigating only the effects of temperature and energy on apoptosis, not on sensitization of tumors to chemotherapy and radiotherapy. The radiosensitization effects of temperatures are described in detail in the literature^33^. However, we speculate, and our study confirms, that mEHT has an additional effect as the heating achieved during mEHT is mild, and despite this, recent clinical results are showing improved outcomes^25,30,34^. We speculate that the energy input and non-thermal field effect of the treatment adds to the tumour killing. In our study, this may be due to the increased apoptosis rates seen with the increase in power when the amplitude modulated field is applied. Murine and human studies have shown an increase in temperature of an average of 2 degrees above normal temperature using mEHT^35^, however the temperatures are not as high as what is achieved during other heating techniques. The clinical results of mEHT do not match the results observed *in vitro*. The limited of clinical level 1 evidence should also be noted, however, this is an area which is growing^31,32,35^. Recently, mEHT was investigated in a Phase III randomized controlled trial, as a sensitizer for CCRT for the treatment of local advance cervical cancer (LACC) patients^30^. The authors claim this was the largest randomized controlled trial of any form of hyperthermia, combining CCRT, for LACC^30^. Improved local disease control and six month local disease free survival was significantly increased with the addition of mEHT^30^ and a preliminary analysis of the survival rates showed a significant improvement in two year survival and two year disease free survival in the sample treated with mEHT^36^. This study shows mEHT does have clinical benefit for cancer patients, even in high risk patients, under resource constrained settings. However, there is always a need to improve the therapeutic effect. Better understanding of the effects of hyperthermia and more specifically of mEHT, as a whole is needed, including optimal protocol development, timing, temperature, temperature monitoring techniques, delivery methods etc.

Hyperthermia therapies are commonly applied in short sessions, about an hour, inducing tissue temperatures from 40 to 44 °C^10^. It has been reported that cell survival at slightly elevated temperatures not only depend on temperature, but also on the exposure time^37^. Our study further adds a parameter, that of energy dosage, to the effect of hyperthermia on cell survival. The energy and treatment time of mEHT has been tested in previous studies^4,15,17,20^. It has been reported that 30 minutes of mEHT treatment time was enough to produce biological response^13,16,18,20,38–40^. In this study, we hoped to shed light on protocols which could achieve better clinical results to match our experimental observation. From our findings, we conclude that higher powers could achieve better therapeutic effect than relatively lower power. This high power was shown to increase the temperature elevation of the cell bag. Temperature elevation from 37 °C to 42 °C by high power, done twice in succession, was shown to be the most effective setting to obtain apoptosis. In a previous publication, we shared results of experiments using various forms of hyperthermia, along with water bath and 8 MHz radiofrequency hyperthermia, where we found mEHT could induce apoptosis during temperature escalation, while other methods could not. Those results indicated that temperature escalation was not the cause of apoptosis. Our belief then was that energy delivery by mEHT was the cause of apoptosis, and temperature escalation was merely a consequence of that energy delivery^18^. However, the temperature elevation may indicate the absorption of energy during mEHT treatment. In clinical application, the temperature elevation is generally accepted to be low and is not even measured during mEHT treatment. Our results indicate that mEHT treatments combined with thermometry, may verify the absorption of energy, until the vender is able to propose another method to check the energy absorption. Having said this, thermometry has shown to be challenging in general practice and the use of thermometry may only be of benefit in a research setting in order to optimize protocols which could be applied in general clinical practice. This study raises another potential protocol, being that power is applied and paused and reapplied, and this could be investigated in murine models for efficacy. Patient tolerance still plays a major role in the ability to achieve higher power outputs and patient tolerance is therefore an important safety parameter used when treating deep seated tumours in which intra-tumoural temperature measurement is not achievable. Another research question is the effect of modulation during the mEHT treatments. The amplitude modulation is a characteristic unique to mEHT treatments which may contribute to the improved results seen at lower temperatures and power outputs as is seen when comparing the results of Harima *et al*. using 8 MHz at a higher power output and a higher temperature, but with non-significant results, and the results of Minnaar *et al*. using mEHT at a maximum power output of 130 W, and with significant results, when treating LACC patients wih CCRT+ /− capacitive heating or mEHT respectively. A follow up study could include a similar design to the present study, but divided into groups with modulation and without modulation.

Highly-localized heating, referred to as nano-heating, is an abstract concept and very difficult to demonstrate. In another recently published study, our team showed gold nanoparticles may prevent mEHT-induced cell death instead of enhancing the cell killing effect of mEHT^41^. Our conclusion from that study was that we did not find that extracellular gold nanoparticles played a role on mEHT’s selectivity and killing of malignant cells. Conversely, cell-incorporated gold nanoparticles reduced cell selectivity and acted as protectors in mEHT treatment. These observations were consistent with previous publications^42,43^. These papers found the nano-heating theory to be very difficult to prove, and that nanoparticles did not help to enhance or prove the nano-heating theory. The role that gold nanoparticles played in the prevention of mEHT-induced cell death may be due to the change of the membrane potential. Hyperthermia induces changes in the configuration of cell membrane proteins, while also altering the membrane transportation of potassium, calcium, and sodium ions^44^, membrane potential^45^, and cellular function^46^. The hyperthermia-resistant cells manifested a significantly higher (up to 15%) membrane potential than the naive cells^47^. The electric current generated from the mEHT tended to flow predominantly through the tumour lesion due to the relatively higher ionic conductivity and abnormal membrane potential of the malignant cells. The changes in membrane potential induced by the incorporated gold nanoparticles may diminish the cell selectivity of the RF current between the normal and malignant cells in mEHT treatment. This may be due to the lower proportion of apoptotic cells after mEHT treatment when cells were preincubated with gold nanoparticles for several hours. Thus, our aim, in this present study, was not to demonstrate the nano-heating phenomenon, but rather to investigate which parameters are the most important factors affecting mEHT-induced apoptotic cell death effect on cancer cells. This may provide hints for the clinical application of mEHT to achieve better clinical outcomes. However, our study only indicated *in vitro* benefits of mEHT. Although temperature increase is just a consequence of energy delivery and is not directly correlated to apoptosis, we do not discard the potential of the elevation of temperature to stimulate the immune system and contribute to the therapeutic benefit (e.g. induce specific T-cell response and abscopal effect) in addition to apoptosis in *in vitro* system.

In conclusion, the results of these experiments show that the effect of mEHT-induced apoptosis was dependent on power dose, based on isothermal conditions. The results imply that mEHT may trigger the stimulation of death receptors to process the apoptotic pathway. One possible reason for the increased rates of apoptosis seen with increased periods of power input is the deposition of energy at the membrane with the occurrence of non-thermal effects. Since there was only two degree Celsius difference in temperature, this difference in general may not be sufficient to achieve the stimulation of death receptors to process apoptotic pathway. The modulated-induced chemical imbalance in cell environment may be a suitable explanation for the apoptotic effect which revealed by Wust *et al*.^48^. The large dose of power at the high-power setting, and large accumulation of this power over a long period of time, induced the greatest rate of cell death. This phenomenon further confirmed our previous findings that mEHT expressed effects not limited to those that are thermal-related, which we had not found with other methods of hyperthermia. However, we did not have ability to collect evidence for an intracellular temperature rise. The only biological effect we observed in this study was the apoptotic cell death effect after mEHT treatment. The induction of apoptotic cell death may be correlated with the intracellular temperature rise, but the evidence is not straight forward. This heterogeneous intracellular temperature distribution during mEHT treatment needs further investigation. We hope our results can provide some opportunity to design future trials. This novel finding warrants further study into the concept of adjusting power dosage during clinical hyperthermia treatment.
